# Long-term renal outcomes comparison between patients with chronic kidney disease and hepatorenal syndrome after living donor liver transplantation

**DOI:** 10.3389/fsurg.2023.1116728

**Published:** 2023-04-03

**Authors:** Hsiang-Yu Tseng, Yu-Hung Lin, Chih-Che Lin, Chao-Long Chen, Chee-Chien Yong, Li-Man Lin, Chih-Chi Wang, Yi-Chia Chan

**Affiliations:** ^1^ Liver Transplantation Center, and Department of Surgery, Kaohsiung Chang Gung Memorial Hospital, and Chang Gung University College of Medicine, Kaohsiung, Taiwan; ^2^ Department of Early Childhood Care and Education, Cheng Shiu University, Kaohsiung, Taiwan

**Keywords:** chronic kidney disease (CKD), end-stage liver disease (ESRD), hepatorenal syndrome (HRS), liver transplantation (LT), living donor LT (LDLT)

## Abstract

**Background and aims:**

Hepatorenal syndrome (HRS) is a disastrous renal complication of advanced liver disease with a poor prognosis. Restoring normal liver function through liver transplantation (LT) is a standardized treatment with favorable short-term survival. However, the long-term renal outcomes in patients with HRS receiving living donor LT (LDLT) are controversial. This study aimed to investigate the prognostic impact of LDLT in patients with HRS.

**Methods:**

We reviewed adult patients who underwent LDLT between July 2008 and September 2017. Recipients were classified into 1) HRS type 1 (HRS1, *N* = 11), 2) HRS type 2 (HRS2, *N* = 19), 3) non-HRS recipients with pre-existing chronic kidney disease (CKD, *N* = 43), and 4) matched normal renal function (*N* = 67).

**Results:**

Postoperative complications and 30-day surgical mortality were comparable among the HRS1, HRS2, CKD, and normal renal function groups. The 5-year survival rate was >90% and estimated glomerular filtration rate (eGFR) transiently improved and peaked at 4 weeks post-transplantation in patients with HRS. However, renal function deteriorated and resulted in CKD stage ≥ III in 72.7% of HRS1 and 78.9% of HRS2 patients (eGFR <60 ml/min/1.73 m^2^). The incidence of developing CKD and end-stage renal disease (ESRD) was similar between the HRS1, HRS2, and CKD groups, but significantly higher than that in the normal renal function group (both *P* < 0.001). In multivariate logistic regression, pre-LDLT eGFR <46.4 ml/min/1.73 m^2^ predicted the development of post-LDLT CKD stage ≥ III in patients with HRS (AUC = 0.807, 95% CI = 0.617–0.997, *P* = 0.011).

**Conclusions:**

LDLT provides a significant survival benefit for patients with HRS. However, the risk of CKD stage ≥ III and ESRD among patients with HRS was similar to that in pre-transplant CKD recipients. An early preventative renal-sparing strategy in patients with HRS is recommended.

## Introduction

Hepatorenal syndrome (HRS) is a functional renal failure that occurs in patients with decompensated cirrhosis, acute-on-chronic liver failure, or acute liver failure. Approximately 20%–40% of advanced cirrhotic patients with acute kidney injury (AKI) develop HRS (1). According to the severity and disease progression rate, the International Club of Ascites (ICA) classifies HRS into types 1 and 2 (1). HRS indicates an extremely poor prognosis in patients with advanced liver disease (2). Liver transplantation (LT) is the definitive treatment for HRS. In addition to restoring liver function and eliminating portal hypertension, renal function also improves after LT because of a reduction in kidney vasoconstriction (2, 3). A systemic review reported that renal dysfunction was reversible in 83% of patients with HRS, but the mortality rate was still higher than that in patients without HRS after LT (4).

Organ shortage and high mortality on the LT waiting list make living donor LT (LDLT) an attractive alternative for patients with HRS (5). Unlike deceased donor LT (DDLT), LDLT can provide timely surgery if a suitable living liver donor is available. However, LDLT is a complex, technically demanding procedure, and a smaller liver volume may increase the risk of post-transplant liver dysfunction and perioperative complications in critically ill patients (6). Kenneth et al. demonstrated that patients with HRS had poor post-LDLT renal function and worse 5-year survival than those of non-HRS patients (78% vs. 95%, *P* = 0.001) (7). However, a recent cohort study showed that the post-LT 5-year survival was similar between HRS and non-HRS patients (54% vs. 63%, *P* = 0.351) (8).

The aforementioned literature discloses the controversial renal function recovery and survival in patients with HRS after LT. Patients with HRS have impaired pre-transplant renal function that is similar to that in patients with chronic kidney disease (CKD). However, unlike long-term renal dysfunction in CKD, HRS is a relatively AKI that occurs in end-stage liver disease. The overall and renal outcomes of patients with HRS undergoing LDLT have not been studied comprehensively. This study thus aimed to investigate the post-LDLT renal function evolution and overall survival of patients with HRS by comparing them to recipients with pre-transplant CKD or normal renal function.

## Patients and methods

### Patient selection

We retrospectively reviewed the medical records of 924 adult recipients of LDLT at Kaohsiung Chang Gung Memorial Hospital between July 2008 and September 2017. The study was approved by the institutional review board (number:202200107B0).

### Definitions of clinical diagnoses

HRS was defined following the diagnostic criteria published by the ICA: (1) cirrhosis and ascites; (2) diagnosis of AKI according to the ICA criteria; (3) no response after two consecutive days of diuretic withdrawal and plasma volume expansion with albumin; (4) absence of shock; (5) no current or recent use of nephrotoxic drugs; and (6) no macroscopic signs of structural kidney injury, which is indicated by proteinuria, microhematuria, or abnormal findings on renal ultrasonography (9). The initial criteria were revised in 2007 and 2015 and subsequently updated in 2019 (1). All patients with HRS included in this study fit the diagnostic criteria described by the ICA; the definition of type-1 HRS is now referred to as HRS-AKI and be defined based on serum creatinine ≥0.3 mg/dl within 48 h or ≥50% from baseline value according to ICA consensus document and/or urinary output ≤0.5 ml/kg body weight ≥6 h. Whereas type-2 HRS is now known as HRS-CKD and be defined as eGFR <60 ml/min/1.73 m^2^ for ≥3 months in the absence of other causes (2).. (Table 1) According to the criteria, the patients were divided into the HRS1 group (11 patients) and the HRS2 group (19 patients). In addition to the HRS1 and HRS2 groups, we included two control groups: the CKD group (43 patients) which consisted of patients diagnosed with CKD stage ≥ III (determined by the estimated glomerular infiltration rate (eGFR) using the Modification of Diet in Renal Disease (MDRD) equation before LDLT) (10) and the normal renal function group (67 patients) which consisted of patients with normal pre-LDLT renal function (determined using propensity score matching based on sex and age). CKD stages ≥ III were defined as eGFR <60 ml/min/1.73 m^2^ 3 months or kidney damage 3months per the Kidney Disease Outcomes Quality Initiative guidelines (11). To distinguish between HRS2 and CKD need to rule out structural kidney injury, proteinuria and microhematuria were routinely checked in our patients. However, for patients with advanced cirrhosis or hepatorenal syndrome, the risk of renal biopsy is relatively high. For such reason, we did not obtain renal biopsy in our recipients. The differential diagnosis was mainly done by the timing of renal deterioration (clinical course) and the duration of renal dysfunction.

**Table 1:** The definition of HRS and CKD.

	Definition
Type 1 HRS (HRS-AKI)	a) Increase in serum creatinine ≥0.3 mg/dl within 48 h or b) Urinary output ≤0.5 ml/kg body weight ≥6 h or c) ≥ 50% increase in serum creatinine from baseline within 3 months
Type 2 HRS (HRS-CKD)	a) eGFR <60 ml/min/1.73 m^2^ for ≥3 months in the absence of other (structural) causes b) < 50% increase in serum creatinine from baseline within 3 months
CKD	a) Kidney damage for ≥3 months, as defined by structural or functional abnormalities of the kidney, with or without decreased eGFR or b) eGFR <60 ml/min/1.73 m^2^ for ≥3 months, with or without kidney damage.

CKD, chronic kidney disease; eGFR, estimated glomerular filtration rate; HRS: hepatorenal syndrome.

### Preoperative preparation

Patients with liver cirrhosis who were diagnosed with HRS and developed more than one organ failure were admitted to the liver intensive care unit before LDLT. The first aim was to evaluate and treat the reversible etiology of renal injury such as gastrointestinal bleeding, sepsis, and spontaneous bacterial peritonitis (3). Additionally, exposure to nephrotoxic agents was avoided. Examples of these include nonsteroidal anti-inflammatory medications, antibiotics with renal toxicity, and radiocontrast. In patients with HRS, vasoconstrictors (e.g., terlipressin) and albumin are used, and precise fluid management is evaluated by continued central venous pressure and urine output monitoring (7). Renal replacement therapy (RRT) is indicated when pulmonary edema, severe hyperkalemia, metabolic acidosis, and/or complications of uremia develop (2). There was no RRT for HRS patients before LDLT in this study. The preoperative eGFR was routinely measured within one week before transplant, and the preoperative eGFR value was used as the baseline eGFR for further comparison.

### Liver transplantation

All LDLTs in this study were ABO-compatible, and partial liver grafts were obtained from living relatives following the Organ Transplant Act of Taiwan. All operations were approved by the Institutional Ethics Committee. Both recipient and donor surgeries followed the protocol used in our liver transplant center, as described elsewhere (12). The graft-to-recipient weight ratio (GRWR) threshold was > 0.8% and the graft-to-recipient standard liver volume ratio was > 40% (12).

### Post-transplant management

Post-LDLT management followed the protocol established in our center (13). Briefly, basiliximab was administered 6 h after portal vein reperfusion (day 0) and on postoperative day 4. Prostaglandin E1 and heparin were started once bleeding tendency was at an acceptable point for 2 weeks. Prophylactic antibiotics including antibacterial and antifungal agents are routinely administered. Hyperimmune cytomegalovirus (CMV) globulin was routinely administered once a week for 1 month to prevent CMV infection. Hepatitis B immunoglobulin (HBIG) and lamivudine were administered to prevent hepatitis B recurrence or *de novo* hepatitis B infection. Nephrotoxic agents such as aminoglycosides were avoided. The details of this process are described later in this text (6, 14). The maintenance immunosuppressive drugs were tacrolimus and mycophenolate mofetil, mainly used in stable recipients (15). Given the significant nephrotoxic effects of calcineurin inhibitors, renal-sparing regimens were used to preserve renal function in patients with HRS (3). Tacrolimus administration was delayed until renal function improved, as evidenced by adequate urine output (1 ml/kg/hour) and decreased serum creatinine levels (14). A mammalian target of rapamycin (mTOR) inhibitor was added or replaced with tacrolimus in patients with impaired renal function (16). Liver ultrasonography, blood biochemistry, including eGFR, and immunosuppression serum levels were routinely followed up every 2–3 months (6). Additionally, regular follow-up by the nephrologist is essential for patients with significant CKD risk factors, such as diabetes and hypertension (14, 17). The ESRD was defined as eGFR <15 ml/min/1.73 m^2^ and the time of RRT was determined by the nephrologist. The last follow-up in this study ended in August 2022, and the minimum follow-up period was 5 years.

### Assessment of outcomes

Primary outcomes were eGFRs measured using the MDRD equation (10) preoperatively and at 1 week, 1 month, 3 months, 6 months, 1 year, 2 years, and 5 years post-LT. Compared to other formulae, the MDRD equation is a more precise and accessible method for measuring renal function in LT recipients (18). Secondary outcomes were the incidence of CKD (defined as eGFR falling below 60 ml/min/1.73 m^2^ according to the KDIGO guideline) at 5 years post-LDLT (11).

### Statistical analysis

Data were collected and analyzed using IBM SPSS version 20 (IBM Corporation, Armonk, NY, United States). Categorical variables between the groups were compared using the Chi-square test. Numerical variables are expressed as medians with interquartile ranges (IQR) and compared using the Kruskal-Wallis test. The change in eGFR (mean ± 2 standard deviations) was calculated using the generalized estimating equation. Univariate and multivariate logistic regression analyses were used to determine the risk factors for post-LT permanent CKD in patients with HRS. Variables with *P* values less than 0.2 were considered significant in the univariate analysis and were included in the multivariate analysis. The preoperative cutoff values of eGFR to determine the prognostic value of post-LT CKD in patients with HRS were calculated using a receiver operating characteristic curve. Kaplan-Meier survival curves and log-rank tests were used to evaluate the 5-year survival. Statistical significance was set at *p* < 0.05.

## Results

### Demographics

A total of 115 (82.1%) males and 25 (17.9%) females were included in this study. The median age was 53–58 years in each group (Table 2). According to the diagnostic criteria (Table 1), 11 patients were classified into the HRS1 group, 19 into the HRS2 group, and 43 into the CKD group. Sixty-seven patients with preoperative normal renal function were identified by propensity score 1:1 matching method and then defined as the reference group. None of the patients were lost to follow-up in this study.

**Table 2:** Characteristics of the patients undergoing LDLT sub-grouped by preoperative renal function.

	Study group				*P*-value
	Reference group: eGFR ≥ 60 ml/min/1.73^2^ (*N* = 67)	HRS1 (*N* = 11)	HRS2 (*N* = 19)	CKD eGFR < 60 ml/min/1.73^2^ (*N* = 43)	Overall
Age (years)	53 (35–68)	53 (45–63)	53 (42–63)	58 (33–66)	0.055
Male sex, *n* (%)	56 (83.6%)	11 (100%)	15 (78.9%)	33 (76.7%)	0.298
BW (kg)	67.6 (58.2–75.2)	75.3 (63.2–88.4)	69.4 (62–77.8)	65.9 (56.9–71.9)	0.084
Child-Pugh score	8 (5–14)	12 (9–15)	11 (8–14)	9 (5–14)	<0.001
MELD score	12 (6–36)	36 (12–42)	22 (9–36)	17 (11–48)	0.001
MELD score ≥30, *n* (%)	6 (9.0%)	7 (58.3%)	5 (26.3%)	7 (16.3%)	<0.001
Primary liver disease, *n* (%)					
Hepatitis B virus	37 (55.2%)	8 (66.7%)	8 (42.1%)	16 (37.2%)	0.152
Hepatitis C virus	19 (28.4%)	0 (0%)	6 (31.6%)	10 (23.3%)	0.177
Alcohol abuse	6 (9.0%)	3 (25.0%)	5 (26.3%)	10 (23.3%)	0.113
Others	5 (5.5%)	1 (8.3%)	0 (0%)	7 (16.3%)	0.191
HCC positive	36 (53.7%)	0 (0%)	3 (15.8%)	16 (37.2%)	<0.001
Preoperative comorbidities, *n* (%)					
Diabetes mellitus	14 (10.8%)	2 (16.6%)	7 (36.8%)	15 (34.8%)	0.087
Hypertension	5 (7.5%)	2 (16.6%)	8 (42.1%)	15 (34.8%)	0.001
Preoperative laboratory variables					
Albumin (g/dl)	3.1 (2.8–3.4)	2.86 (2.5–3.5)	2.97 (2.7–3.3)	2.9 (2.7–3.2)	0.344
Total bilirubin (mg/dl)	2.0 (1.3–2.7)	29.7 (4.5–38.2)	10.9 (1.2–26)	1.8 (1.0–10.2)	0.007
Creatinine (mg/dl)	0.68 ± 0.14	2.52 ± 2.20	1.60 ± 0.90	2.10 ± 1.35	<0.001
eGFR (ml/min/1.73 m^2^)	112 (102–129)	24 (17–26)	46 (24–69)	38 (22–46)	<0.001
Perioperative variables					
Ascites (ml)	0 (0–1500)	3,900 (2550–7200)	5,200 (3900–13,350)	3,600 (350–9800)	<0.001
Blood loss (ml)	2,300 (1200–4800)	7,000 (1400–16,200)	4,650 (2700–9500)	5,000 (2150–8000)	0.003
GRWR	0.98 (0.86–1.14)	0.89 (0.87–1.08)	0.91 (0.80–1.15)	0.90 (0.82–1.08)	0.670
Warm ischemia time (min)	47 (39–52)	50 (47–56)	39 (32–46)	41 (37–49)	0.004
Cold ischemia time (min)	45 (33–59)	42 (33–47)	43 (33–56)	46 (31–61)	0.594
Operation time (min)	630 (567–694)	567 (498–584)	605 (563–650)	580 (545–660)	0.054
Post-op complication ≥ Gr. IIIb, *n* (%)	28 (41.7%)	6 (50.0%)	5 (26.3%)	25 (58.1%)	0.083
30-day surgical mortality, *n* (%)	0 (0%)	0 (0%)	0 (0%)	0 (0%)	1.000
Tacrolimus as initial CNI, *n* (%)	64 (95.5%)	12 (100.0%)	17 (89.4%)	41 (95.3%)	0.891
mTOR conversion, *n* (%)	26 (38.8%)	11 (91.6%)	15 (78.9%)	37 (86.0%)	<0.001
Follow up (years)	9.9 (9.6–12)	7.5 (5.8–9.3)	9.0 (5.8–10.3)	8.6 (5.5–10.8)	0.189

*P*-value are expressed as median (interquartile range) or number (percentage).

BW, body weight; CKD, chronic kidney disease; CNI, calcineurin inhibitor; eGFR, estimated glomerular filtration rate; GRWR, graft to recipient weight ratio; HCC, Hepatocellular carcinoma; HRS, hepatorenal syndrome; LDLT, living donor liver transplantation; MELD, model for end stage liver disease; mTOR, mammalian target of rapamycin.

The prevalence of underlying liver diseases was as follows: hepatitis B (48.9%), hepatitis C (24.8%), alcoholic liver disease (17.0%), and other etiologies (9.3%). There were significantly fewer patients with HCC in HRS1 (*n* = 0, 0%) and HRS2 (*n* = 3, 15.8%) than in the CKD (*n* = 16, 37.2%) and reference groups (*n* = 36, 53.7%) (*P* = 0.018 and 0.001, respectively). There were no differences in age, sex, primary liver disease, or pre-existing comorbidities, including hypertension and diabetes, among the four groups (*P* > 0.05). However, the severity of liver dysfunction, in terms of Child-Pugh score, MELD score, and serum total bilirubin level, was significantly higher in the HRS1 and HRS2 groups than in the CKD and reference groups (*P* < 0.05).

All three groups (HRS1, HRS2, and CKD) had significantly lower eGFR and higher serum creatinine levels than those of the reference group (Table 2). In addition, the HRS1 group had worse renal function than the HRS2 group did. Nonetheless, none of the patients with HRS required RRT before LDLT. Interval time from onset of HRS1 to transplantation was showed in Table 3.

**Table 3:** Interval time from onset of HRS1 to transplantation.

HRS1 Patient list	Date of renal function deterioration	Date of LDLT	Interval (days)
1	12-Dec-2008	14-Dec-2008	2
2	23-Mar-2011	27-Mar-2011	4
3	24-Feb-2014	04-Mar-2014	8
4	12-Mar-2014	15-Mar-2014	6
5	02-Jun-2014	12-Jun-2014	10
6	30-Jan-2015	01-Feb-2015	1
7	04-Mar-2016	05-Mar-2016	1
8	20-Sep-2012	27-Sep-2012	7
9	26-Aug-2014	28-Aug-2014	3
10	07-May-2016	17-May-2016	10
11	21-Jun-2016	28-Jun-2016	7

HRS, hepatorenal syndrome; LDLT, living donor liver transplantation.

The perioperative parameters, including cold/warm ischemia time, GRWR, and intraoperative blood loss (7000 vs. 4,650 vs. 5,000 vs. 2300 ml; *P* = 0.891), did not show statistical differences among the HRS1, HRS2, CKD, and reference groups. The ascites amount at the time of LT showed significant differences among 4 groups (*P* < 0.01) but no statistical differences among the HRS1, HRS2, CKD (*P* = 0.159) (Table 2). The risk of postoperative Clavien-Dindo complications ≥ grade IIIb (59% vs. 26.3% vs. 58.1% vs. 41.7%; *P* = 0.073) was similar in the HRS1, HRS2, CKD, and reference groups. No 30-day postoperative mortalities were observed.

More than 90% of the patients were administered tacrolimus. As expected, mTOR was more frequently prescribed to patients with renal insufficiency (91.6% for HRS1, 78.9% for HRS2, 86.0% for CKD, and 38.8% for the reference group, *P* < 0.001; Table 2). The details between each groups were present in Supplementary Material Table S1.

### Renal function after LDLT

**Figure 1:**
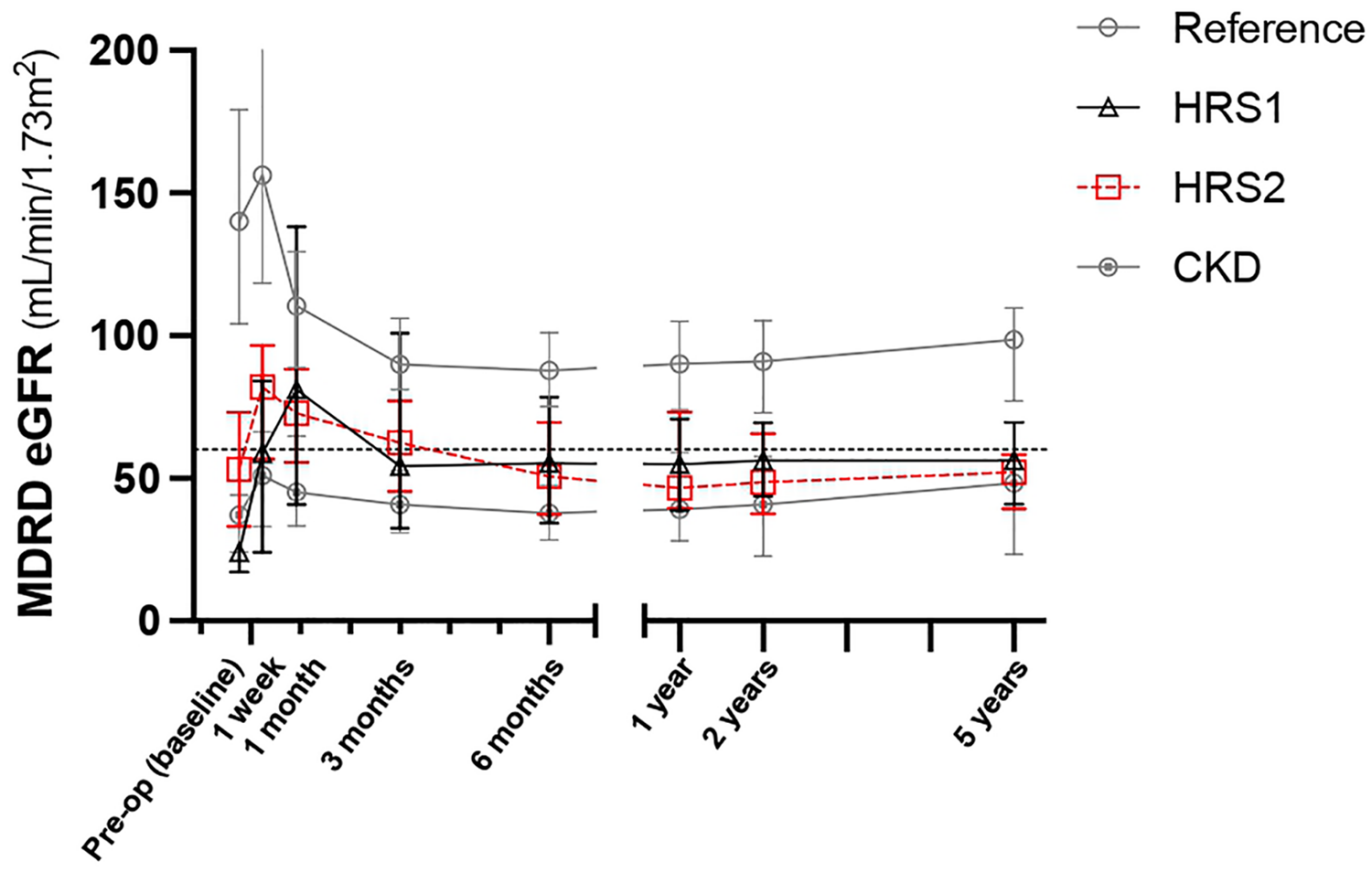
Serial change of eGFR in five years after LDLT. Values are expressed as median ± interquartile range. CKD, chronic kidney disease; eGFR, estimated glomerular filtration rate; HRS, hepatorenal syndrome; LDLT, living donor liver transplantation.

The eGFR of the reference group declined gradually 1 month after LT and stabilized 1 year after LDLT (Table 4). We further compared the eGFR between the HRS1, HRS2, and CKD groups. One week after LT, the eGFR (58, 82, and 51 ml/min/1.73 m^2^, respectively) markedly improved comparing to the preoperative values in all three groups. One month after transplantation, the eGFR in HRS1 continued to improve, whereas in HRS2 and CKD, the eGFR declined (Table 4). However, at the third month of transplantation, the eGFR (54, 62, and 41 ml/min/1.73 m^2^, respectively) deteriorated in all three groups. Between the 3rd and 6th months after LT, the eGFR of HRS2 kept decreasing whereas the eGFR of HRS1 and CKD groups maintained at relatively levels. One year after transplantation, eGFR stabilized. There was no significant difference in eGFR in the HRS1, HRS2, and CKD groups at the first year post-LT (55, 47, and 39 ml/min/1.73 m^2^, respectively; *P* = 0.098), second year (56, 49, and 41 ml/min/1.73 m^2^, respectively; *P* = 0.082), and fifth year (56, 52, and 48 ml/min/1.73 m^2^, respectively; *P* = 0.149). The pre-LT and early post-LT eGFR among the three groups were significantly different (all *P* < 0.05), but became similar 1 year after LT (all *P* > 0.05). Interestingly, despite HRS1 started with the worst pretransplant eGFR among all the groups, the eGFR could be maintained at around 55 ml/min/1.73 m^2^ from the 3rd month after LT throughout the 5-year follow-up (Figure 1).

**Table 4:** Series of eGFR (ml/min/1.73 m^2^) after LDLT in four groups stratified by preoperative renal function.

	Study group				*P*-value
	Reference: eGFR ≥ 60 ml/min/1.73 m^2^ (*N* = 67)	HRS1 (*N* = 11)	HRS2 (*N* = 19)	CKD: eGFR < 60 ml/min/1.73^2^ (*N* = 43)	Overall
Pre-operation	112 (102–129)	24 (17–26)	46 (24–69)	38 (22–46)	<0.001
Post-LT 1 week	156 (118–223)	58 (21–79)	82 (57–97)	51 (33–66)	<0.001
Post-LT 1 month	110 (88–128)	68 (36–131)	74 (55–89)	47 (32–65)	<0.001
Post-LT 3 month	90 (81–106)	54 (32–101)	62 (45–77)	41 (31–53)	<0.001
Post-LT 6 month	88 (75–101)	55 (34–78)	51 (37–70)	38 (28–48)	<0.001
Post-LT 1 year	91 (74–105)	55 (39–73)	47 (39–73)	39 (28–59)	<0.001
Post-LT 2 years	91 (73–105)	56 (44–69)	49 (38–66)	41 (23–58)	<0.001
Post-LT 5 years	99 (77–110)	56 (41–70)	52 (39–58)	48 (24–56)	<0.001

Values are expressed as median (interquartile range).

CKD, chronic kidney disease; eGFR, estimated glomerular filtration rate; HRS, hepatorenal syndrome; LDLT, living donor liver transplantation.

CKD stage ≥ III developed in 8 (72.7%), 15 (78.9%), 39 (90.7%), and 5 (7.5%) patients in the HRS1, HRS2, CKD, and reference groups (respectively) after 5 years of LDLT (*P* = 0.001). Moreover, 12 (27.9%) patients in the CKD group eventually progressed to the ESRD status, while one (8.3%) patient in the HRS1 group, and two (10.5%) patients in the HRS2 group required RRT (*P* = 0.248). Finally, one patient (8.3%) in the HRS1 group and three patients (6.9%) in the CKD group underwent kidney transplantation at the end of follow-up (*P* = 0.293) (Table 5).

**Table 5:** Post-LDLT 5th year renal outcome in four groups stratified by preoperative renal function.

	Study group				*P*-value
	Reference: eGFR ≥ 60 ml/min/1.73 m^2^ (*N* = 67)	HRS1 (*N* = 11)	HRS2 (*N* = 19)	CKD: eGFR < 60 ml/min/1.73 m^2^ (*N* = 43)	Overall
Post-op 5^th^ year renal outcome					
CKD stage ≥ III, *n* (%)	5 (7.5%)	8 (72.7%)	15 (78.9%)	39 (90.7%)	<0.001
ESRD: eGFR < 15 ml/min/1.73 m^2^					
Hemodialysis, *n* (%)	0 (0%)	0 (0%)	2 (10.5%)	9 (23.1%)	<0.001
Kidney transplantation, *n* (%)	0 (0%)	1 (8.3%)	0 (0.0%)	3 (6.9%)	0.158
5-year survival rate	89.6%	91.7%	94.7%	81.4%	0.587
Cause of death					
Infection, *n* (%)	2 (28.6%)	0 (0%)	1 (100%)	5 (62.5%)	0.817
HCC, *n* (%)	5 (71.4%)	0 (0%)	0 (0%)	0 (0%)	0.125
Other, *n* (%)	0 (0%)	1 (100%)*	0 (0%)	3 (37.5%)^@^	0.552

*cardiovascular event; ^@^one of each cardiovascular event, veno-occlusive disease and unknown.

CKD, chronic kidney disease; eGFR, estimated glomerular filtration rate; ESRD, end-stage renal disease; HRS, hepatorenal syndrome; LDLT, living donor liver transplantation.

### Cause of death and five-year survival

**Figure 2:**
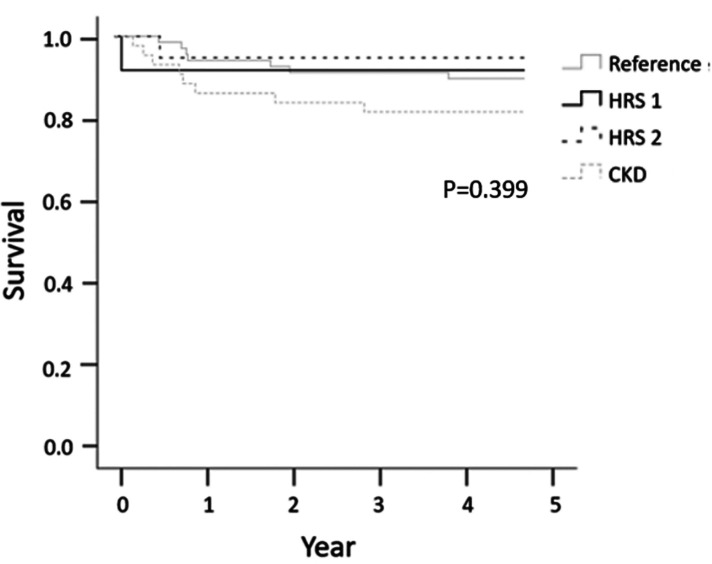
Five-year survival according to preoperative renal function, categorized by HRS1, HRS2, CKD, and reference groups.

The 5-year survival rates after LDLT were 89.6%, 91.7% for HRS1, 94.7% for HRS2, and 81.4% for CKD group, respectively. There was no significant difference between the groups (*P* = 0.399) (Figure 2). Infection was the most common cause of mortality in the CKD group (*n* = 5). HCC progression was the most common cause of mortality in the reference group (*n* = 5) (Table 5).

### Risk factors of permanent CKD in HRS patients undergoing LDLT

**Figure 3:**
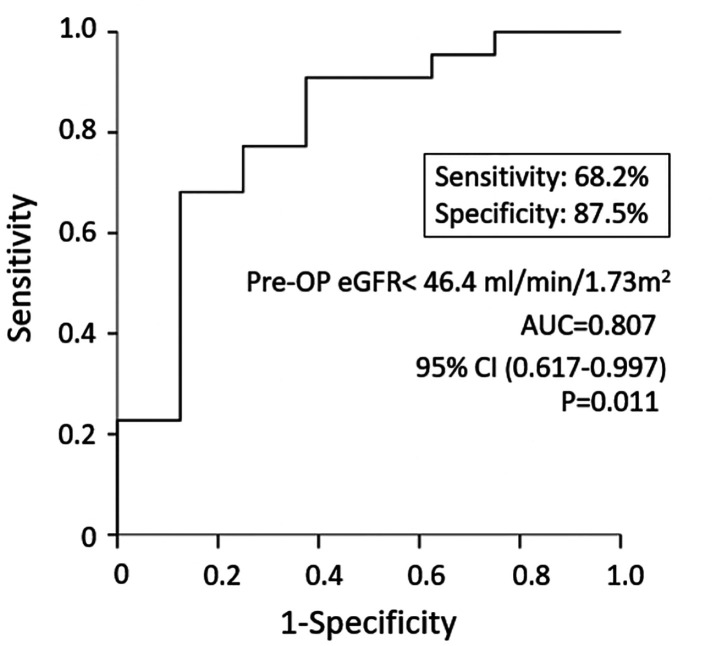
Preoperative eGFR predict permanent CKD in LDLT recipients with HRS.

In a univariate analysis (Table 6), the variables significantly associated with CKD stage ≥ III after 5 years of LDLT included pre-LT eGFR (odds ratio [OR] = 0.96, 95% confidence interval [CI] = 0.920–0.995, *P* = 0.028) and postoperative complications ≥ Gr.IIIb (OR = 0.16, 95% CI = 0.025–0.999, *P* = 0.050). MELD score, pre-LT creatinine, pre-LT eGFR, GRWR, post-LDLT complication ≥ Gr. IIIb, and use of terlipressin were entered into multivariate logistic regression. The significant variable in the multivariate analysis was the pre-LT eGFR (OR = 0.96, 95% CI = 0.920–0.995, *P* = 0.028). When the cutoff value of pre-LT eGFR was set at <46.4 ml/min/1.73 m^2^ (AUC = 0.807, 95% CI = 0.617–0.997, *P* = 0.011), the sensitivity and specificity for predicting CKD stage ≥ III after 5 years of LDLT were 68.2% and 87.5%, respectively (Figure 3).

**Table 6:** Risk factors of post-LT permanent CKD for HRS patients undergoing LDLT.

	Univariate			Multivariate		
	OR	95% Cl	*P*-value	OR	95% Cl	*P*-value
Age (years)	0.96	0.821–1.115	0.574			
Male sex	0.91	0.080–10.210	0.935			
BW (kg)	1.00	0.938–1.073	0.933			
Child-Pugh score	1.22	0.764–1.932	0.411			
MELD score	1.06	0.972–1.148	0.194			
MELD score ≥30	7.00	0.734–66.801	0.091			
HCC positive	0.70	0.055–8.966	0.784			
Diabetes mellitus	3.27	0.334–31.914	0.309			
Hypertension	0.78	0.144–4.212	0.771			
Albumin (g/dl)	1.06	0.202–5.550	0.945			
Total bilirubin (mg/dl)	1.00	0.954–1.052	0.935			
Pre-LDLT Creatinine (mg/dl)	5.49	0.995–30.312	0.051			
Pre-LDLT eGFR (ml/min/1.73 m^2^)	0.96	0.920–0.995	0.028	0.96	0.920–0.995	0.028
Terlipressin use	0.27	0.027–2.614	0.256			
Pre-LDLT shock	0.70	0.055–8.966	0.784			
Pre-LDLT HE	0.50	0.095–2.628	0.413			
Pre-LDLT respiratory failrue	1.56	0.147–16.455	0.714			
ACLF	2.41	0.456–12.720	0.301			
Blood loss (ml)	1.00	1.000–1.000	0.345			
GRWR	0.03	0.000–2.354	0.111			
Post-op complication. ≥ Gr. IIIb	0.16	0.025–0.999	0.050			
Tacrolimus	3.00	0.165–54.566	0.458			
mTOR conversion	4.00	0.447–35.788	0.215			

ACLF, acute-on-chronic liver failure; BW, body weight; CKD, chronic kidney disease; eGFR, estimated glomerular filtration rate; HCC, hepatocellular carcinoma; HE, hepaticencephalopathy; HRS, hepatorenal syndrome; LDLT, living donor liver transplantation; MELD, model for end stage liver disease; mTOR, mammalian target of rapamycin.

## Discussion

Preoperative renal dysfunction in LT recipients has been associated with poor renal outcomes and overall survival (8). Several retrospective studies have reported that kidney function significantly improves after LT in patients with HRS, irrespective of whether it is a DDLT or LDLT (19, 20). Our study included 30 patients with HRS who underwent LDLT and compared the long-term renal outcomes and survival of these patients to that of patients with preoperative CKD and normal renal function. One of our findings was that renal function improved within one month after LDLT but declined gradually in both HRS1, HRS2, and CKD patients. The eGFR can not become normal after 5-year follow up, but the eGFR still much improved especially in HRS1 patients. Also, our results showed that permanent CKD stage ≥ III developed in more than 70% of patients with HRS; ESRD occurred in almost 10% of patients with HRS in the 5-year follow-up. The renal outcomes in patients with HRS, defined as CKD and ESRD, were similar to those in pre-LT CKD recipients. In addition, pre-LT eGFR <46.4 ml/min/1.73 m^2^ was a prognostic factor for permanent CKD in our study. Finally, patients with HRS showed a non-inferior 5-year survival rate (>90%) compared to patients with normal renal function and CKD.

A previous meta-analysis reported that an increased risk of CKD frequently exists among LT recipients with reduced renal function prior to transplant (17). A retrospective matched case-control study showed that HRS2 was an independent predictor of post-transplant CKD stage III at 3 months after transplantation (21). Another retrospective study analyzed patients with HRS1 and showed that the incidence of developing CKD beyond stage III at 12 months was highest in the advanced stages of HRS-AKI (22). Hence, we investigated whether preoperative kidney function, either acute or chronic renal failure, has an impact on adverse renal events. Corresponding to other studies, our results demonstrated that preoperative impaired renal function, including HRS and CKD, contributes to a higher risk of progressive post-LT renal dysfunction. Another interesting study by Thomas et al. reported that improvement in preoperative renal function was not associated with better post-LT renal outcomes or survival (8). Therefore, pre-transplant acute or chronic kidney injury, even with post-LDLT transient improvement, plays an important role in the long-term prognosis.

Complete recovery of renal function after LT in patients with HRS is rare (22, 23). In a previous study, treatment with terlipressin reduced the risk of CKD after LT in patients with HRS-AKI (22, 24). However, Thomas et al. found that stabilization of renal function following terlipressin administration prior to LT did not result in better renal outcomes in patients with HRS (8). In coherence with our findings, terlipressin use was not a protective factor against long-term CKD development in patients with HRS in the multivariate logistic regression.

Calcineurin inhibitors (CNI) are widely used as the main immunosuppressants in LT. The use of CNI in patients undergoing LT is associated with a higher incidence of CKD development (25). Therefore, several strategies have been implemented to lower the risk for post-transplant CKD in patients undergoing LT, including the early switch to mTOR (25, 26) and reduction of CNI dosage (27). In our current practice, in patients with renal dysfunction risk factors, we reduced the CNI dosage and administered an mTOR inhibitor early to prevent renal function deterioration. Recent studies revealed that tacrolimus use did not increase the risk of CKD after LT in patients with HRS-AKI (21, 22, 28). Therefore, the early introduction of mTOR inhibitors as a renal-sparing strategy is still under investigation.

ESRD in LT recipients is strongly correlated with poor quality of life, elevated cardiovascular events, risk of liver graft dysfunction, and higher mortality (13, 29). In our study, approximately 10% of patients with HRS and 28% of patients with CKD progressed to ESRD within 5 years after LT. Although no specific risk factor to predict the occurrence of ESRD was identified in this study, previous studies have reported that pre-LT eGFR lesser than 60 ml/min/1.73 m^2^ and diabetes were associated with progression to ESRD after LT (18, 30). The lower the eGFR after LT, the sooner the development of renal failure occurred (23). For LT recipients with postoperative ESRD, renal transplantation is recommended based on proven better outcomes compared to those remaining on dialysis (31, 32).

A systematic review and meta-analysis showed that the all–cause mortality at 1,3, and 5 years post-LT tended to be higher among patients with HRS than among non-HRS patients. However, most of the included studies were DDLT (4). Moreover, previous cohort studies have reported that patients with HRS had worse survival than non-HRS patients after LDLT (31, 33). Thomas et al. demonstrated that poor renal function prior to LT was a strong predictor of worse post-LT survival (8). Fangcong et al. revealed that survival was worse in patients with pre-LT advanced-stage HRS and post-LT RRT (22). In contrast, in this study, patients with HRS who underwent LDLT had a satisfactory survival rate that was comparable to that of the reference group.

This study had several limitations. First, it was a single-center study, and our results require further validation in other centers. Second, the eGFR was retrospectively calculated using the MDRD equation; although adjusted for age and sex, it only represents some determinants of renal function. Although the true GFR may be overestimated in cirrhotic patients with reduced muscle mass, eGFR determined by MDRD remains a convenient method for clinical use (30). Third, HRS may occur in patients with preoperative CKD, the so-called acute-on-chronic renal failure. However, no patients with HRS were diagnosed with CKD before LT in this cohort, and these patients may require more attention in future studies. Finally, the small sample size might have caused inevitable biases in the results; therefore, a large prospective study is required to confirm these findings.

## Conclusions

In our study, the renal function much improved after LDLT especially in HRS type 1 patients. The significant survival rate and improvement of renal function makes LDLT is a safe and effective treatment option for HRS patients. However, long-term renal dysfunction remains problematic for these patients. Early renal-sparing strategies to preventrenal function deterioration and kidney transplants for patients progressing to ESRD are recommended to achieve a better long-term quality of life.

## Data Availability

The original contributions presented in the study are included in the article/**Supplementary Material**, further inquiries can be directed to the corresponding author.

## References

[1] Angeli P Garcia-Tsao G Nadim MK Parikh CR. News in pathophysiology, definition and classification of hepatorenal syndrome: a step beyond the international club of ascites (ICA) consensus document. J Hepatol. (2019) 71(4):811–22. 10.1016/j.jhep.2019.07.002 31302175

[2] Ojeda-Yuren AS Cerda-Reyes E Herrero-Maceda MR Castro-Narro G Piano S. An integrated review of the hepatorenal syndrome. Ann Hepatol. (2021) 22:100236. 10.1016/j.aohep.2020.07.008 32846202

[3] Tariq R Singal AK. Management of hepatorenal syndrome: a review. J Clin Transl Hepatol. (2020) 8(2):192–9. 10.14218/JCTH.2020.00011 32832400 PMC7438356

[4] Utako P Emyoo T Anothaisintawee T Yamashiki N Thakkinstian A Sobhonslidsuk A. Clinical outcomes after liver transplantation for hepatorenal syndrome: a systematic review and meta-analysis. Biomed Res Int. (2018) 2018:5362810. 10.1155/2018/5362810 29992152 PMC5994306

[5] Goldaracena N Marquez M Selzner N Spetzler VN Cattral MS Greig PD Living vs. Deceased donor liver transplantation provides comparable recovery of renal function in patients with hepatorenal syndrome: a matched case-control study. Am J Transplant. (2014) 14(12):2788–95. 10.1111/ajt.12975 25277134

[6] Wang YC Yong CC Lin CC Alam H Naseer F Lin YH Excellent outcome in living donor liver transplantation: treating patients with acute-on-chronic liver failure. Liver Transpl. (2021) 27(11):1633–43. 10.1002/lt.26096 33977657

[7] Chok KS Fung JY Chan SC Cheung TT Sharr WW Chan AC Outcomes of living donor liver transplantation for patients with preoperative type 1 hepatorenal syndrome and acute hepatic decompensation. Liver Transpl. (2012) 18(7):779–85. 10.1002/lt.23401 22290625

[8] Horvatits T Hubener P Touma M Horvatits K Fischer L Lohse AW Improvement of renal function prior to liver transplantation is not associated with better long-term renal outcome or survival. Ann Hepatol. (2021) 26:100559. 10.1016/j.aohep.2021.100559 34656773

[9] Angeli P Gines P Wong F Bernardi M Boyer TD Gerbes A Diagnosis and management of acute kidney injury in patients with cirrhosis: revised consensus recommendations of the international club of ascites. Gut. (2015) 64(4):531–7. 10.1136/gutjnl-2014-308874 25631669

[10] Levey AS Greene T Schluchter MD Cleary PA Teschan PE Lorenz RA Glomerular filtration rate measurements in clinical trials. Modification of diet in renal disease study group and the diabetes control and complications trial research group. J Am Soc Nephrol. (1993) 4(5):1159–71. 10.1681/ASN.V451159 8305642 PMC2866096

[11] Ikizler TA Burrowes JD Byham-Gray LD Campbell KL Carrero JJ Chan W KDOQI Clinical practice guideline for nutrition in CKD: 2020 update. Am J Kidney Dis. (2020) 76(3 Suppl 1):S1–S107. 10.1053/j.ajkd.2020.03.008 32829751

[12] Chen CL Fan ST Lee SG Makuuchi M Tanaka K. Living-donor liver transplantation: 12 years of experience in Asia. Transplantation. (2003) 75(3 Suppl):S6–11. 10.1097/01.TP.0000046533.93621.C7 12589130

[13] Chan YC Yeh CH Li LC Chen CL Wang CC Lin CC Excess risk of Major adverse cardiovascular and kidney events after acute kidney injury following living donor liver transplantation. J Clin Med. (2022) 11(11):3100. 10.3390/jcm11113100 PMC9181469 35683487

[14] Lin CC Chuang FR Lee CH Wang CC Chen YS Liu YW The renal-sparing efficacy of basiliximab in adult living donor liver transplantation. Liver Transpl. (2005) 11(10):1258–64. 10.1002/lt.20520 16184544

[15] Wu YJ Lin YH Yong CC Li WF Wang SH Wang CC Safe one-to-one dosage conversion from twice-daily to once-daily tacrolimus in long-term stable recipients after liver transplantation. Ann Transplant. (2016) 21:30–4. 10.12659/AOT.895118 26782179

[16] Geissler EK Schnitzbauer AA Zulke C Lamby PE Proneth A Duvoux C Sirolimus use in liver transplant recipients with hepatocellular carcinoma: a randomized, multicenter, open-label phase 3 trial. Transplantation. (2016) 100(1):116–25. 10.1097/TP.0000000000000965 26555945 PMC4683033

[17] Fabrizi F Dixit V Martin P Messa P. Pre-transplant kidney function predicts chronic kidney disease after liver transplant: meta-analysis of observational studies. Dig Dis Sci. (2011) 56(5):1282–9. 10.1007/s10620-010-1529-2 21221799

[18] Bahirwani R Campbell MS Siropaides T Markmann J Olthoff K Shaked A Transplantation: impact of pretransplant renal insufficiency. Liver Transpl. (2008) 14(5):665–71. 10.1002/lt.21367 18433034

[19] Demirbas BT Piskin T Dayangac M Yaprak O Oklu L Yuzer Y Successful treatment of severe hepatorenal syndrome with living donor liver transplantation. Hepatogastroenterology. (2012) 59(119):2305–6. 10.5754/hge10791 23435146

[20] Lee JP Kwon HY Park JI Yi NJ Suh KS Lee HW Clinical outcomes of patients with hepatorenal syndrome after living donor liver transplantation. Liver Transpl. (2012) 18(10):1237–44. 10.1002/lt.23493 22714872

[21] Tan HK Marquez M Wong F Renner EL. Pretransplant type 2 hepatorenal syndrome is associated with persistently impaired renal function after liver transplantation. Transplantation. (2015) 99(7):1441–6. 10.1097/TP.0000000000000557 25643142

[22] Li F Wang T Zhan L Jia Z Luo T Chen S Clinical outcomes of liver transplantation in patients with hepatorenal syndrome: a single center study in China. Front Surg. (2021) 8:781648. 10.3389/fsurg.2021.781648 35155548 PMC8831834

[23] Sanchez EQ Melton LB Chinnakotla S Randall HB McKenna GJ Ruiz R Predicting renal failure after liver transplantation from measured glomerular filtration rate: review of up to 15 years of follow-up. Transplantation. (2010) 89(2):232–5. 10.1097/TP.0b013e3181c42ff9 20098288

[24] Piano S Gambino C Vettore E Calvino V Tonon M Boccagni P Response to terlipressin and albumin is associated with improved liver transplant outcomes in patients with hepatorenal syndrome. Hepatology. (2021) 73(5):1909–19. 10.1002/hep.31529 32870499

[25] Lin YH Lin CC Wang CC Wang SH Liu YW Yong CC The 4-week serum creatinine level predicts long-term renal dysfunction after adult living donor liver transplantation. Transplant Proc. (2012) 44(3):772–5. 10.1016/j.transproceed.2012.03.034 22483492

[26] Teperman L Moonka D Sebastian A Sher L Marotta P Marsh C Calcineurin inhibitor-free mycophenolate mofetil/sirolimus maintenance in liver transplantation: the randomized spare-the-nephron trial. Liver Transpl. (2013) 19(7):675–89. 10.1002/lt.23658 23775875

[27] Boudjema K Camus C Saliba F Calmus Y Salame E Pageaux G Reduced-dose tacrolimus with mycophenolate mofetil vs. Standard-dose tacrolimus in liver transplantation: a randomized study. Am J Transplant. (2011) 11(5):965–76. 10.1111/j.1600-6143.2011.03486.x 21466650

[28] Maurel P Premaud A Carrier P Essig M Barbier L Rousseau A Evaluation of longitudinal exposure to tacrolimus as a risk factor of chronic kidney disease occurrence within the first-year post-liver transplantation. Transplantation. (2021) 105(7):1585–94. 10.1097/TP.0000000000003384 32639405

[29] Zand MS Orloff MS Abt P Patel S Tsoulfas G Kashyap R High mortality in orthotopic liver transplant recipients who require hemodialysis. Clin Transplant. (2011) 25(2):213–21. 10.1111/j.1399-0012.2010.01238.x 20331690

[30] Longenecker JC Estrella MM Segev DL Atta MG. Patterns of kidney function before and after orthotopic liver transplant: associations with length of hospital stay, progression to End-stage renal disease, and mortality. Transplantation. (2015) 99(12):2556–64. 10.1097/TP.0000000000000767 25989501

[31] Bittermann T Abt PL Olthoff KM Kaur N Heimbach JK Emamaullee J. Impact of advanced renal dysfunction on posttransplant outcomes after living donor liver transplantation in the United States. Transplantation. (2021) 105(12):2564–70. 10.1097/TP.0000000000003728 33660658 PMC8410875

[32] Horvatits T Pischke S Proske VM Fischer L Scheidat S Thaiss F Outcome and natural course of renal dysfunction in liver transplant recipients with severely impaired kidney function prior to transplantation. United Eur Gastroenterol J. (2018) 6(1):104–11. 10.1177/2050640617707089 PMC5802668 29435320

[33] Okamura Y Hata K Inamoto O Kubota T Hirao H Tanaka H Influence of hepatorenal syndrome on outcome of living donor liver transplantation: a single-center experience in 357 patients. Hepatol Res. (2017) 47(5):425–34. 10.1111/hepr.12764 27323334

