# Supplementary material for: Long-term renal outcomes comparison between patients with chronic kidney disease and hepatorenal syndrome after living donor liver transplantation
Source: Front Surg. 2023 Apr 3;10:1116728. doi: 10.3389/fsurg.2023.1116728 (PMC10106629; doi:10.3389/fsurg.2023.1116728)
Supplement: Supplementary file 1 [file Datasheet1.pdf]

Supplementary Table 1. Characteristics of the patients undergoing LDLT sub-grouped by preoperative renal function

	Study group				P-value						
	Reference group: eGFR ≥ 60 ml/min/1.73² (N=67)	HRS1 (N=11)	HRS2 (N=19)	CKD eGFR < 60 ml/min/1.73² (N=43)	Overall	Ref. vs. HRS1	Ref. vs HRS2	Ref. vs. CKD	HRS1 vs. HRS2	HRS1 vs. CKD	HRS2 vs. CKD
Age (years)	53 (35-68)	53 (45-63)	53 (42-63)	58 (33-66)	0.055	1.000	1.000	0.054	1.000	0.547	0.323
Male sex, n (%)	56 (83.6%)	11 (100%)	15 (78.9%)	33 (76.7%)	0.298	1.000	1.000	1.000	0.899	0.451	1.000
BW (kg)	67.6 (58.2-75.2)	75.3 (63.2-88.4)	69.4 (62-77.8)	65.9 (56.9-71.9)	0.084	0.179	1.000	1.000	0.800	0.072	1.000
Child-Pugh score	8 (5-14)	12 (9-15)	11 (8-14)	9 (5-14)	<0.001	0.001	0.005	0.421	1.000	0.031	0.326
MELD score	12 (6-36)	36 (12-42)	22 (9-36)	17 (11-48)	0.001	0.000	0.006	0.002	0.011	0.001	1.000
MELD score ≥30, n (%)	6 (9.0%)	7 (58.3%)	5 (26.3%)	7 (16.3%)	<0.001	<0.001	0.494	1.000	0.062	0.002	1.000
Primary liver disease, n (%)											
Hepatitis B virus	37 (55.2%)	8 (66.7%)	8 (42.1%)	16 (37.2%)	0.152	1.000	1.000	0.399	1.000	0.714	1.000
Hepatitis C virus	19 (28.4%)	0 (0%)	6 (31.6%)	10 (23.3%)	0.177	0.274	1.000	1.000	0.335	0.681	1.000
Alcohol abuse	6 (9.0%)	3 (25.0%)	5 (26.3%)	10 (23.3%)	0.113	0.806	0.457	0.314	1.000	1.000	1.000
Others	5 (5.5%)	1 (8.3%)	0 (0%)	7 (16.3%)	0.191	1.000	1.000	1.000	1.000	1.000	0.259
HCC positive	36 (53.7%)	0 (0%)	3 (15.8%)	16 (37.2%)	<0.001	0.003	1.000	0.422	1.000	0.114	0.575
Preoperative comorbidities, n (%)											
Diabetes mellitus	14 (10.8%)	2 (16.6%)	7 (36.8%)	15 (34.8%)	0.087	1.000	0.999	0.363	0.594	0.368	1.000
Hypertension	5 (7.5%)	2 (16.6%)	8 (42.1%)	15 (34.8%)	0.001	1.000	0.007	0.003	0.757	1.000	1.000
Preoperative laboratory variables											
Albumin (g/dL)	3.1 (2.8-3.4)	2.86 (2.5-3.5)	2.97 (2.7-3.3)	2.9 (2.7-3.2)	0.541	1.000	1.000	1.000	1.000	1.000	1.000
Total bilirubin (mg/dL)	2.0 (1.3-2.7)	29.7 (4.5-38.2)	10.9 (1.2-26)	1.8 (1.0-10.2)	0.007	<0.001	0.246	0.591	0.597	0.007	1.000
Creatinine (mg/dL)	0.68 ± 0.14	2.52 ± 2.20	1.60 ± 0.90	2.10 ± 1.35	<0.001	<0.001	<0.001	<0.001	1.000	1.000	0.778
eGFR (ml/min/1.73 m²)	112 (102-129)	24 (17-26)	46 (24-69)	38 (22-46)	<0.001	<0.001	<0.001	<0.001	0.412	1.000	0.770
Perioperative variables											
Ascites (ml)	0 (0-1500)	3900 (2550-7200)	5200 (3900-13350)	3600 (350-9800)	<0.001	0.002	0.000	0.000	1.349	1.000	0.323
Blood loss (ml)	2300 (1200-4800)	7000 (1400-16200)	4650 (2700-9500)	5000 (2150-8000)	0.057	0.144	1.000	0.287	1.000	1.000	1.000
GRWR	0.98 (0.86-1.14)	0.89 (0.87-1.08)	0.91 (0.80-1.15)	0.90 (0.82-1.08)	0.670	1.000	1.000	1.000	1.000	1.000	1.000
Warm ischemia time (min)	47 (39-52)	50 (47-56)	39 (32-46)	41 (37-49)	0.004	1.000	0.007	0.995	0.017	0.556	0.217
Cold ischemia time (min)	45 (33-59)	42 (33-47)	43 (33-56)	46 (31-61)	0.594	1.000	1.000	1.000	1.000	1.000	1.000
Operation time (min)	630 (567-694)	567 (498-584)	605 (563-650)	580 (545-660)	0.054	0.068	1.000	0.857	1.000	0.646	1.000
Post-op complication ≥ Gr. IIIb, n (%)	28 (41.7%)	6 (50.0%)	5 (26.3%)	25 (58.1%)	0.083	1.000	1.000	0.555	0.802	1.000	0.125
30-day surgical mortality, n (%)	0 (0%)	0 (0%)	0 (0%)	0 (0%)	1.000	1.000	1.000	1.000	1.000	1.000	1.000
Tacrolimus as initial CNI, n (%)	64 (95.5%)	12 (100.0%)	17 (89.4%)	41 (95.3%)	0.891	1.000	1.000	1.000	1.000	1.000	1.000
mTOR conversion, n (%)	26 (38.8%)	11 (91.6%)	15 (78.9%)	37 (86.0%)	<0.001	0.006	0.003	0.001	1.000	1.000	1.000
Follow up (years)	9.9 (9.6-12)	7.5 (5.8-9.3)	9.0 (5.8-10.3)	8.6 (5.5-10.8)	0.189	0.849	0.084	0.061	0.841	1.000	1.000

P-value are expressed as median (interquartile range) or number (percentage).

BW: body weight; CKD: chronic kidney disease; CNI: calcineurin inhibitor; eGFR: estimated glomerular filtration rate;

GRWR: graft to recipient weight ratio; HCC: Hepatocellular carcinoma; HRS: hepatorenal syndrome; LDLT: living donor liver transplantation;

MELD: model for end stage liver disease; mTOR: mammalian target of rapamycin
