# Supplementary material for: Long-term renal outcomes comparison between patients with chronic kidney disease and hepatorenal syndrome after living donor liver transplantation
Source: Front Surg. 2023 Apr 3;10:1116728. doi: 10.3389/fsurg.2023.1116728 (PMC10106629; doi:10.3389/fsurg.2023.1116728)
Supplement: Supplementary file 2 [file Datasheet2.pdf]

Supplementary Table 2. Series of eGFR (ml/min/1.73m^2) after LDLT in four groups stratified by preoperative renal function

	Study group				P-value						
	**Reference: eGFR ≥ 60 ml/min/1.73m^2 (N=67)**	**HRS1 (N=11)**	**HRS2 (N=19)**	**CKD: eGFR < 60 ml/min/1.73^2 (N=43)**	**Overall**	**Ref. vs. HRS1**	**Ref. vs HRS2**	**Ref. vs. CKD**	**HRS1 vs. HRS2**	**HRS1 vs. CKD**	**HRS2 vs. CKD**
Pre-operation	112 (102-129)	24 (17-26)	46 (24-69)	38 (22-46)	<0.001	<0.001	<0.001	<0.001	0.107	0.958	0.012
Post-LT 1 week	156 (118-223)	58 (21-79)	82 (57-97)	51 (33-66)	<0.001	<0.001	<0.001	<0.001	0.886	0.879	0.063
Post-LT 1 month	110 (88-128)	68 (36-131)	74 (55-89)	47 (32-65)	<0.001	0.187	<0.001	<0.001	0.782	0.144	0.012
Post-LT 3 month	90 (81-106)	54 (32-101)	62 (45-77)	41 (31-53)	<0.001	0.242	<0.001	<0.001	0.961	0.405	0.285
Post-LT 6 month	88 (75-101)	55 (34-78)	51 (37-70)	38 (28-48)	<0.001	0.006	<0.001	<0.001	0.970	0.181	0.041
Post-LT 1 year	91 (74-105)	55 (39-73)	47 (39-73)	39 (28-59)	<0.001	<0.001	<0.001	<0.001	0.999	0.383	0.290
Post-LT 2 years	91 (73-105)	56 (44-69)	49 (38-66)	41 (23-58)	<0.001	0.003	<0.001	<0.001	0.983	0.331	0.321
Post-LT 5 years	99 (77-110)	56 (41-70)	52 (39-58)	48 (24-56)	<0.001	0.003	<0.001	<0.001	0.999	0.511	0.296

Values are expressed as median (interquartile range)

CKD: chronic kidney disease; eGFR: estimated glomerular filtration rate; HRS: hepatorenal syndrome;

LDLT: living donor liver transplantation
